# Incidence of Diabetes Mellitus and Associated Factors in the Era of Antiretroviral Drugs With a Low Metabolic Toxicity Profile

**DOI:** 10.1093/ofid/ofae112

**Published:** 2024-03-01

**Authors:** Maria Luisa Montes, Carmen Busca, Nuria Espinosa, José Ignacio Bernardino, Sofia Ibarra-Ugarte, Luz Martín-Carbonero, Cristina Moreno, Juan Macias, Antonio Rivero, Miguel Cervero-Jiménez, Juan González-García

**Affiliations:** Unidad VIH, Servicio de Medicina Interna, Hospital Universitario La Paz, Institute for Health Research, Madrid, Spain; Centre for Biomedical Research on Infectious Diseases (CIBERINFEC), Instituto de Salud Carlos III, Madrid, Spain; Unidad VIH, Servicio de Medicina Interna, Hospital Universitario La Paz, Institute for Health Research, Madrid, Spain; Centre for Biomedical Research on Infectious Diseases (CIBERINFEC), Instituto de Salud Carlos III, Madrid, Spain; Infectious Diseases and Microbiology Clinical Unit, Instituto de Biomedicina de Sevilla, University Hospital Virgen del Rocío, CSIC, Universidad de Sevilla, Seville, Spain; Unidad VIH, Servicio de Medicina Interna, Hospital Universitario La Paz, Institute for Health Research, Madrid, Spain; Centre for Biomedical Research on Infectious Diseases (CIBERINFEC), Instituto de Salud Carlos III, Madrid, Spain; Servicio de Enfermedades Infecciosas, Hospital Universitario Basurto, Bilbao, Spain; Unidad VIH, Servicio de Medicina Interna, Hospital Universitario La Paz, Institute for Health Research, Madrid, Spain; Centre for Biomedical Research on Infectious Diseases (CIBERINFEC), Instituto de Salud Carlos III, Madrid, Spain; National Center for Epidemiology, Instituto de Salud Carlos III, Madrid, Spain; Centre for Biomedical Research on Infectious Diseases (CIBERINFEC), Instituto de Salud Carlos III, Madrid, Spain; Departamento de Medicina, IBiS, Universidad de Sevilla, Hospital Universitario Virgen de Valme, Seville, Spain; Centre for Biomedical Research on Infectious Diseases (CIBERINFEC), Instituto de Salud Carlos III, Madrid, Spain; Department of Infectious Diseases, Hospital Universitario Reina Sofia, Instituto Maimonides de Investigación Biomédica de Córdoba, Universidad de Córdoba, Cordoba, Spain; Servicio de Medicina Interna, Hospital Universitario Severo Ochoa, Leganés, Spain; Unidad VIH, Servicio de Medicina Interna, Hospital Universitario La Paz, Institute for Health Research, Madrid, Spain; Centre for Biomedical Research on Infectious Diseases (CIBERINFEC), Instituto de Salud Carlos III, Madrid, Spain

**Keywords:** DM2, incidence, treatment naive, ART, rilpivirine

## Abstract

**Objective:**

The incidence of type 2 diabetes mellitus (T2DM) has risen dramatically. Among people living with HIV (PLHIV), chronic disease (now >15 cases/1000 in the general population worldwide) and long-term exposure to antiretroviral therapy (ART) can alter metabolic processes early, favoring insulin resistance and T2DM. We retrospectively studied the incidence of T2DM and associated factors in the Cohort of the Spanish AIDS Research Network, a prospective cohort of PLHIV enrolled at diagnosis and before initiation of ART.

**Methods:**

PLHIV were aged >18 years and ART naive at inclusion. The incidence of new diagnoses of T2DM after initiation of ART (per 1000 person-years) was calculated. Predictors of a diagnosis of T2DM were identified by a Cox proportional hazards model adjusted for statistically significant and clinically relevant variables.

**Results:**

Cumulative incidence was 5.9 (95% CI, 5.1–6.7) per 1000 person-years, increasing significantly in persons aged >50 years to 14.4 (95% CI, 10.4–19.3). Median time to diagnosis of T2DM was 27 months. Only age and higher education were significant. Interestingly, higher education was associated with a 33% reduction in the incidence of T2DM. Having received tenofovir disoproxil fumarate + (lamivudine or emtricitabine) + rilpivirine was almost significant as a protective factor (hazard ratio, 0.49; 95% CI, .24–1.01; *P* = .05).

**Conclusions:**

The incidence of T2DM in PLHIV in Spain was high, especially in persons aged >50 years. Age was the factor most closely associated with onset, and educational level was the factor most associated with reduced risk. We highlight the lack of association between HIV-related factors and T2DM and show that, within nonnucleoside reverse transcriptase inhibitors, rilpivirine could prove more benign for metabolic comorbidities.

## Teaser

The incidence of type 2 diabetes mellitus is high in people living with HIV in Spain. Age was the factor most closely associated with onset of type 2 diabetes mellitus. Rilpivirine could prove more benign in lipid-related comorbidities and glucose-related metabolic comorbidities.

The metabolic conditions affecting people living with HIV (PLHIV) have played a key role in the care of this population since the initial stages of triple antiretroviral therapy (ART) [1–3]. Lipid metabolism abnormalities and concern over the associated consequences for morbidity and mortality in PLHIV in the medium and long terms have given rise to a considerable number of cohort studies, clinical trials, and clinical practice guidelines. However, more recently, other highly concerning comorbid conditions being researched include glucose metabolism abnormalities, onset of type 2 diabetes mellitus (T2DM), and the association between T2DM and metabolic liver disease, with many questions remaining unanswered [4, 5].

The incidence of T2DM among the general public in developed and developing countries has risen dramatically, now standing at >15 cases per 1000 worldwide [6]. This finding will have a huge impact on public health in the coming decades. The main risk factors are age, overweight/obesity, socioeconomic level, and changes in dietary habits [6–8]. Moreover, chronic inflammatory processes and various types of drugs (eg, immunosuppressive and immunomodulatory agents) alter carbohydrate metabolism and increase insulin resistance [9, 10]. Therefore, PLHIV share characteristics with the general population and persons with chronic diseases. In addition, they are exposed long-term to antiretroviral drugs that can alter metabolic processes early in adipose tissue and muscle and liver tissue, thus favoring insulin resistance and T2DM [11–13].

In 2018, Nansseu et al [14] performed a systematic review and meta-analysis of the global incidence of and factors associated with T2DM in PLHIV. The authors examined the results of studies published during the last 10 years in populations from throughout the world and found that the incidence and cumulative incidence of T2DM differed considerably, thus highlighting the importance of more local analysis of these variables. In Europe, incidence rates range from 3 to 28 per 1000 person-years of follow-up, with notable differences between northern and southern countries (higher in Mediterranean countries) and with respect to the period when ART was taken (eg, before 2010), when the regimens differed considerably from those used today.

In the present study, we analyzed the incidence of T2DM among PLHIV in Spain who initiated ART between January 2010 and December 2019. We also analyzed factors associated with onset of T2DM and the potential effect of initiating ART in a substudy of patients exposed to the same combination of antiretroviral drugs for at least 24 months. The treatments analyzed had a favorable metabolic safety and efficacy profile. We believe that the results that we present paint a clear picture of current risks of T2DM in PLHIV receiving ART.

## METHODS

### Study Design and Setting

Our retrospective observational study was based on data and samples from PLHIV in the Cohort of the Spanish HIV/AIDS Research Network (CoRIS), a prospective cohort of adults who were HIV positive and ART naive at inclusion. CoRIS comprises 46 centers caring for PLHIV in 13 Spanish autonomous communities (regions), ranging from medium-sized local centers to large teaching centers. CoRIS covers the diversity that characterizes PLHIV in Spain today. The cohort has >19 000 patients in follow-up, with loss to follow-up <19% [15].

The CoRIS database collects demographic and clinical data, HIV transmission category, ART history, previous opportunistic diseases, specific non-AIDS diseases, and serologic and immunovirologic data. Newly diagnosed cardiovascular comorbidities are specifically recorded following a well-defined protocol. Since 2014, height and weight have been recorded regularly at recruitment in the cohort and at follow-up visits. For all patients enrolled in the cohort, updated clinical and biological data were requested at 6 ± 2 months. Follow-up was terminated upon death, change to another follow-up center outside the cohort, or failure to attend scheduled visits.

### Participants

To be eligible for this study, participants had to be PLHIV aged >18 years who entered and initiated ART between 1 January 2010 and 30 December 2019 and were not diagnosed with T2DM (fasting blood sugar ≥126 mg/dL or blood sugar ≥200 mg/dL in the oral glucose tolerance test). As of 30 December 2019, data from 16 759 PLHIV were registered in the CoRIS database, and 9153 met inclusion criteria.

### Variables Analyzed

Sociodemographic, epidemiologic, anthropometric, clinical, analytical, and therapeutic variables were analyzed before initiation of ART. We analyzed baseline ART combinations by drug family. In the subanalysis of factors associated with the diagnosis of T2DM, we included the combinations preferred between 2010 and 2019 and used in most patients: tenofovir disoproxil fumarate (TDF) + (lamivudine [3TC] or emtricitabine [FTC]) + efavirenz (EFV), TDF + (3TC or FTC) + rilpivirine (RPV), TDF + (3TC or FTC) + darunavir boosted (DRV/b), abacavir (ABC) + 3TC + dolutegravir (DTG). The analysis was based only on the first ART combination taken uninterrupted for at least 6 months (or the second regimen if the first lasted <6 months).

Participants were followed up from initiation of ART until the last visit or diagnosis of T2DM, as appropriate. Antiretroviral regimens, occurrence of non-AIDS events, concomitant medication, and laboratory parameters were examined at baseline. Deaths and loss to follow-up were recorded until the participant developed T2DM or until the last visit analyzed.

### Main Outcome Measure

The main outcome measure was a diagnosis of T2DM after initiation of ART, as made by the attending physician according to 1 of the following currently accepted definitions:

- Cases recorded as such in the cohort registry
- Participants with at least 2 blood sugar readings >126 mg/dL with an interval ≤12 months between readings
- Participants who had been receiving continuous hypoglycemic therapy on a continuous basis (insulin, metformin, sulfonylureas, glitazones, sodium-glucose cotransporter 2, and glucagon-like peptide 1), excluding those whose received these drugs for management of prediabetes and/or obesity.

The date of diagnosis was considered the earliest of the following: the date when the participant was registered as diabetic, the date when antidiabetic treatment was started, or the second date when the blood sugar reading reached ≥126 mg/dL.

### Independent Variables

The covariates analyzed included the following: sex at birth, age, world region of birth, HIV transmission category (men who have sex with men, intravenous drug user, heterosexual relations, other), time in education, baseline CD4+ T-cell count, months living with HIV, prior AIDS-defining conditions, non-AIDS events before ART, hepatitis C virus (HCV) serologic status, corticosteroid and/or bronchodilator treatment (considering any exposure at initiation of ART recorded in the concomitant medications section of the database), and first ART regimens selected from the main combinations: TDF + (3TC or FTC) + EFV, TDF + (3TC or FTC) + RPV, TDF + (3TC or FTC) + DRVr/c, and ABC + 3TC)+ DTG.

### Statistical Analysis

We reported the sociodemographic and clinical characteristics of the study population at initiation of ART using frequency tables for categorical variables and median and IQRs for continuous variables. Groups were compared by χ^2^ and Fisher exact tests in the case of categorical variables and by unpaired *t* test and Mann-Whitney test in the case of quantitative data.

The incidence rate of new diagnoses of T2DM (per 1000 person-years) was calculated.

Predictors associated with the diagnosis of T2DM were identified by the Cox proportional hazards model and included age, HIV transmission category, time in education, baseline CD4+ T-cell count, months living with HIV, prior AIDS-defining conditions, non-AIDS events before ART, serologic status against HCV, and first ART regimen. An adjusted analysis was performed with statistically significant and clinically relevant variables. A competing risk analysis was performed taking death as a competing event.

We performed a sensitivity analysis after excluding from the global cohort patients with T2DM whose follow-up was <33 months, since this is the value of the upper limit of the 95% CI of the median time to T2DM. Our aim was to reduce the risk that they had not developed T2DM owing to insufficient follow-up time.

For all tests, a 2-tailed *P* value <.05 was considered statistically significant. The analysis was performed with SPSS version 26.0 (IBM Corp).

### Ethics

This study complied with all local legal and ethical requirements. The protocol was approved by the Ethics Committee of Hospital Universitario La Paz, (PR[AG]272-2019). Before inclusion, all the patients in the CoRIS cohort signed an informed consent document for performance of observational studies with the cohort data. The study was conducted according to good clinical practice guidelines and the Declaration of Helsinki.

## RESULTS

### Study Population and Incidence of T2DM

The study population comprised 9153 PLHIV, with a follow-up of 32 803 person-years. Median time from inclusion to initiation of ART was 0.82 months (IQR, 0.2–3.2). We recorded 192 new cases of T2DM over a median follow-up of 40.3 months (IQR, 17.4–65.7). Diagnosis of T2DM was based on registry criteria in 58% of cases, blood sugar in 33%, and hypoglycemic medication in 9%. The baseline characteristics are shown in Table 1.

**Table 1.** Baseline Characteristics of the Overall Cohort (N = 9153)

	Total (N = 9153)	No T2DM (n = 8961)	T2DM (n = 192)	*P* Value
Baseline variables				
Age, y	35.9 (29.2–43.9)	35.7 (29.1–43.6)	46.3 (39–54.4)	<.001
Female sex	1158 (12.7)	1131 (12.6)	27 (14.1)	.51
CD4, cells/µL	368 (212–532)	370 (215–534)	295.5 (105–449)	<.001
Body mass index	23.5 (21.5–25.9)	23.5 (21.5–25.8)	26.6 (23.5–29.6)	<.001
Months between diagnosis of HIV and initiation of ART	2.3 (0.9–9.5)	2.3 (0.9–9.5)	1.7 (0.8–7.6)	.08
Route of infection				
MSM	6103 (69.3)	6006 (69.7)	97 (53.3)	…^a^
IDU	315 (3.6)	299 (3.5)	16 (8.8)	…^a^
Heterosexual relations	2325 (26.4)	2262 (26.2)	63 (34.6)	…^a^
Other	60 (0.7)	54 (0.6)	6 (3.3)	<.001^a^
Origin				
Sub-Saharan Africa	367 (4)	354 (4)	13 (6.8)	…^a^
Spain	5383 (58.8)	5276 (58.9)	107 (55.7)	
Europe	1168 (12.8)	1123 (12.5)	45 (23.4)	…^a^
Latin America/Caribbean	2009 (21.9)	1988 (22.2)	21 (10.9)	
North Africa	96 (1)	94 (1)	2 (1)	
Other	130 (1.4)	126 (1.4)	4 (2.1)	<.001
Longest time in education				
Basic secondary	2429 (32.9)	2349 (32.5)	80 (51)	…^a^
Preuniversity/trade school	2465 (33.4)	2422 (33.5)	43 (27.4)	
University/higher	2485 (33.7)	2451 (33.9)	34 (21.7)	<.001^a^
Before ART				
HCV	544 (6.3)	519 (6.2)	25 (13.5)	<.001
Corticosteroids and/or bronchodilators	68 (0.7)	66 (0.7)	2 (1)	.65
Non–AIDS-related events	552 (6)	543 (6.1)	9 (4.7)	.54
Cardiovascular non–AIDS-related events	60 (0.7)	54 (0.6)	6 (3.1)	<.01
AIDS	925 (10.1)	886 (9.9)	39 (20.3)	<.001
First ART regimen				
2 NRTI + 1 NNRTI	2508 (27.4)	2441 (27.2)	67 (34.9)	…^a^
2 NRTI + 1 PI	1710 (18.7)	1666 (18.6)	44 (22.9)	
2 NRTI + 1 INI	4186 (45.7)	4119 (46)	67 (34.9)	…^a^
Other	749 (8.2)	735 (8.2)	14 (7.3)	.01
Follow-up variables ^b^				
Interruption first ART regimen	5611 (61.3)	5495 (61.3)	116 (60.4)	.82
Time undergoing ART, mo	39 (17–64)	40 (18–65)	27 (8–50)	<.001
Death	200 (2.2)	190 (2.1)	10 (5.2)	.01
Loss to follow-up	667 (7.3)	656 (7.3)	11 (5.7)	.48
Months of follow-up	40 (17–66)	409 (17–65)	65 (41–90)	<.001
Months until diagnosis of T2DM			28 (8–51)	

Total: including time after the diagnosis of T2DM. Data are presented as median (IQR) or No. (%).

Abbreviations: ART, antiretroviral therapy; HCV, hepatitis C virus; IDU, intravenous drug user; INI, integrase inhibitors; MSM, men who have sex with men; NNRTI, nonnucleoside reverse transcriptase inhibitor; NRTI, nucleoside reverse transcriptase inhibitor; PI, protease inhibitor; T2DM, type 2 diabetes mellitus.

^a^Differences (*P* < .05) in multiple comparisons.

^b^Censored at diagnosis of T2DM or at 31 December 2019 if no T2DM.

We observed that patients who developed T2DM during follow-up were older at initiation of ART and had a higher body mass index (BMI). They were also characterized by a lower mean CD4 lymphocyte count and a higher percentage of AIDS diagnoses. The route of HIV transmission differed between groups, with intravenous drug use and heterosexual relations as the main routes in the T2DM group. At diagnosis of HIV infection, a higher percentage of coinfection by HCV was associated with parenteral transmission. The groups did not differ in terms of bronchodilator treatment (n = 55; median, 43 months; IQR, 24–64), oral corticosteroids (n = 13; median, 20 months; IQR, 7–44), or both (n = 7); Table 1.

**Figure 1.**
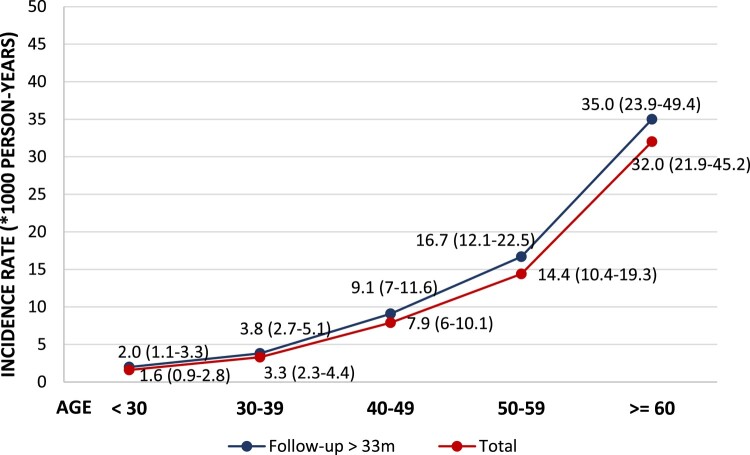
Incidence of new diagnoses of type 2 diabetes mellitus by decade. Data in parentheses indicate 95% CI.

The 192 diagnoses of T2DM accounted for an incidence rate of 5.9 (95% CI, 5.1–6.7) per 1000 patient-years of follow-up. Median time to onset of T2DM was 27 months (95% CI, 22–33; IQR, 8–51) after initiating ART. Incidence grew significantly by age group, with the highest rates reported for individuals aged >50 years (Figure 1).

We recorded losses to follow-up and deaths from baseline to onset of T2DM or the end of follow-up. Losses were 7.3% (95% CI, 6.8%–7.8%), with no significant differences between individuals who developed T2DM and those who did not. The global mortality rate was 2.2% (95% CI, 1.9%–2.5%), although this was higher in those who developed T2DM (5.2% vs 2.1%, *P* = .009). The main causes of death were AIDS-defining events (21%), non–AIDS-defining cancers (16.5%), and non–AIDS-defining infections (27%). Eight deaths from cardiovascular causes were recorded during follow-up (Table 1, Supplementary Table 1).

We performed a second analysis (sensitivity analysis) in which we excluded patients with T2DM whose follow-up time was <33 months (upper limit of the 95% CI of the median time to T2DM) to reduce the risk of them not developing T2DM owing to insufficient follow-up time. A total of 5315 patients were included in this analysis. The general characteristics of this group are shown in Supplementary Table 1. The baseline, sociodemographic, and HIV-related characteristics, as well as differences in these characteristics according to onset or not of T2DM, were similar in this subgroup and in the overall cohort. The incidence of T2DM was 6.8 (95% CI, 5.9–7.9) per 1000 person-years of follow-up, and this increased gradually per decade of age (Figure 1).

### Factors Associated With Development of T2DM

We performed a predictive analysis of the factors associated with development of T2DM. Analysis of all variables with significant differences at baseline showed that those associated with T2DM were age, BMI, heterosexual transmission, time in education, HCV coinfection, non–AIDS-related cardiovascular events, CD4 lymphocyte count, and AIDS stage. The multivariable analysis that was adjusted for all the significant or clinically relevant variables, including CD4 lymphocyte count and AIDS stage, revealed that the only variables that maintained significance were age (hazard ratio, 1.07 for each year; 95% CI, 1.06–1.08; *P* < .001) and time in education >12 years (hazard ratio, 0.676; 95% CI, .45–.96; *P* = .031). The result remained unchanged in a stepwise multivariable analysis (Table 2). BMI was not included in the multivariable analysis owing to the high number of missing values (62.5%).

**Table 2.** Factors Associated With Diagnosis of T2DM, Excuding ART

	Total Cohort				Cohort With Follow-up >33 mo			
	Univariable		Multivariable (n = 7071), HR (95% CI), *P* Value		Univariable		Multivariable (n = 4301), HR (95% CI), *P* Value	
	No.	HR (95% CI), *P* Value	Enter	Final	No.	HR (95% CI), *P* Value	Enter	Final
Age at baseline	8986	1.07 (1.06–1.08), <.001	1.07 (1.05–1.08), <.001	1.07 (1.06–1.08), <.001	5313	1.07 (1.06–1.08), .001	1.07 (1.05–1.08), <.001	1.07 (1.06–1.08), <.001
CD4, cells/mL	8730	1.00 (1.00–1.00), .001	1.00 (1.00–1.00), .949	NS	5162	1.00 (1.00–1.00), .003	1.00 (1.00–1.00), .798	NS
Body mass index	3351	1.14 (1.10–1.19), <.001	…	…	1721	1.16 (1.11–1.21), <.001	…	…
Months with HIV infection	8986	1.00 (.99–1.00), .546	…	…	5315	1.00 (.99–1.00), .418	…	…
MSM	8644	0.52 (.39–.69), <.001	0.97 (.67–1.40), .872	NS	5143	0.52 (.39–.70), <.001	0.96 (.67–1.39), .839	NS
Time in education >12 y	7247	0.55 (.38–.80), .002	0.68 (.46–1.01), .054	0.66 (.45–.96), .031	4411	0.55 (.38–.81), .002	0.69 (.46–1.02), .065	0.67 (.45–.98), .037
Before ART								
HCV	8768	2.14 (1.41–3.26), <.001	1.43 (.86–2.38), .167	NS	5190	2.06 (1.35–3.14), .001	1.40 (.84–2.33), .194	NS
CV NAE	8986	5.12 (2.27–11.54), <.001	1.87 (.74–4.69), .184	NS	5315	5.1 (2.26–11.46), <.001	1.87 (.74–4.69), .185	NS
AIDS	8986	2.15 (1.51–3.05), <.001	1.12 (.70–1.79), .626	NS	5315	2.09 (1.47–2.97), <.001	1.12 (.70–1.78), .628	NS

Abbreviations: ART, antiretroviral therapy; CV, cardiovascular; HCV, hepatitis C virus; HR, hazard ratio; MSM, men who have sex with men; NAE, non-aids events; NS, not significant.

In the subcohort of patients whose follow-up was >33 months, the variables associated with onset of T2DM were equivalent to those of the overall cohort in the univariable and multivariable analyses, with similar HR values.

### Factors Associated With Onset of T2DM, Including ART

We performed a subanalysis to evaluate the possible impact of ART on onset of T2DM. We selected individuals whose first ART regimen remained uninterrupted for at least 6 months (or the second regimen if the first lasted <6 months). The median duration of this regimen was 24 months (IQR, 12–37). The total duration of follow-up in the subcohort was 45 months (95% CI, 22.6–67.8).

A total of 6040 individuals fulfilled this selection criterion. The combinations received were as follows: TDF + (3TC or FTC) + EFV, TDF + (3TC or FTC) + RPV, TDF + (3TC or FTC) + DRVr/c, ABC + 3TC + DTG, TDF + (3TC or FTC) + DTG, TDF + (3TC or FTC) + raltegravir (RAL), or TDF + (3TC or FTC) + elvitegravir/cobicistat (EVG/c). The univariable analysis included the first 4 combinations, since these were the only ones with a greater percentage of patients within the regimens considered preferential in the ART recommendations in force at the time when the regimens were indicated. ART was administered in 1 or 2 combinations in 72% of patients, with no differences between the T2DM and non-T2DM groups (Table 3). The first remained unchanged for a median of 86% (39%–100%) of follow-up.

**Table 3.** Baseline and Follow-up Characteristics of the Population Receiving ART for ≥6 months

	Total (n = 6040)	No T2DM (n = 5913)	T2DM (n = 127)	*P* Value
Baseline variables				
Age, y	35.6 (29.2–43.5)	35.4 (29.0–43.2)	45.5 (39.5–54.2)	<.001
Female sex	700 (11.6)	684 (11.6)	16 (12.6)	.67
CD4, cells/µL	370.0 (218–535)	373 (221–536)	242 (99–449)	<.001
Body mass index	23.5 (21.5–25.7)	23.5 (21.5–25.7)	26.6 (23.5–28.4)	<.001
Months from diagnosis of HIV to initiation of ART	2.6 (1–11)	2.6 (1–11)	1.6 (0.7–7.5)	<.01
Route of infection				
MSM	4095 (70.7)	4030 (71.1)	65 (54.2)	…^a^
IDU	190 (3.3)	181 (3.2)	9 (7.5)	…^a^
Heterosexual relations	1464 (25.3)	1422 (25.1)	42 (35.0)	…^a^
Other	39 (0.7)	35 (0.6)	4 (3.3)	<.001^a^
Origin				
Sub-Saharan Africa	230 (3.8)	221 (3.7)	9 (7.1)	
Spain	3683 (61.0)	3609 (61.0)	74 (58.3)	
Europe	708 (11.7)	681 (11.5)	27 (21.3)	…^a^
Latin America and Caribbean	1279 (21.2)	1266 (21.4)	13 (10.2)	…^a^
North Africa	60 (1.0)	58 (1.0)	2 (1.6)	
Other	80 (1.3)	78 (1.3)	2 (1.6)	<.01
Longest time in education				
Up to secondary	1546 (32.1)	1491 (31.7)	55 (52.9)	…^a^
Up to preuniversity/trade school	1617 (33.6)	1589 (33.7)	28 (26.9)	
University/higher	1650 (34.3)	1629 (34.6)	21 (20.2)	<.001^a^
Before initiation of ART				
HCV	353 (6.2)	336 (6.0)	17 (14.0)	<.01
Corticosteroids and/or bronchodilators	52 (0.9)	50 (0.8)	2 (1.6)	.3
Non–AIDS-related events	371 (6.1)	364 (6.2)	7 (5.5)	1
Cardiovascular non–AIDS-related events	38 (0.6)	34 (0.6)	4 (3.1)	<.01
AIDS before ART	638 (10.6)	610 (10.3)	28 (22.0)	<.001
Class of first ART regimen				
2 NRTI + 1 NNRTI	2093 (34.7)	2044 (34.6)	49 (38.6)	
2 NRTI + 1 PI	874 (14.5)	850 (14.4)	24 (18.9)	
2 NRTI + 1 II	2996 (49.6)	2943 (49.8)	53 (41.7)	
Other	77 (1.3)	76 (1.3)	1 (0.8)	.24
Baseline ART				
TDF + (3TC/FTC)+EFV	1239 (20.5)	1199 (20.3)	40 (31.5)	<.01
TDF + (3TC/FTC)+RPV	910 (15.1)	898 (15.2)	12 (9.4)	.08
TDF + (3TC/FTC)+RAL	444 (7.4)	431 (7.3)	13 (10.2)	.22
TDF + (3TC/FTC)+DRV/b	815 (13.5)	793 (13.4)	22 (17.3)	.19
TDF + (3TC/FTC)+DTG	422 (7.0)	416 (7.0)	6 (4.7)	.38
ABC + 3TC+DTG	1777 (29.4)	1751 (29.6)	26 (20.5)	.02
TDF + (3TC/FTC)+ELV/c	433 (7.2)	425 (7.2)	8 (6.3)	.86
Follow-up variables^b^				
Interruption first ART regimen	3431 (56.8)	3363 (56.9)	68 (53.5)	.47
Time undergoing ART, mo	44 (22–67)	39.7 (18–65)	27 (7.3–53)	.001^a^
No. of ART lines during follow-up				
1	2709 (44.9)	2647 (44.8)	62 (48.8)	
2	1841 (30.5)	1803 (30.5)	38 (29.9)	
3	992 (16.4)	975 (16.5)	17 (13.4)	
≥4	498 (8.2)	488 (8.3)	10 (7.9)	.75

Data are presented as median (IQR) or No. (%).

Abbreviations: 3TC, lamivudine; ABC, abacavir; ART, antiretroviral therapy; DRV, darunavir boosted; DTG, dolutegravir; EFV, efavirenz; EVG/c, elvitegravir/cobicistar; FTC, emtricitabine; HCV, hepatitis C virus; IDU, intravenous drug user; INI, integrase inhibitors; MSM, men who have sex with men; NNRTI, nonnucleoside reverse transcriptase inhibitor; NRTI, nucleoside reverse transcriptase inhibitor; PI, protease inhibitor; RAL, raltegravir; RPV, rilpivirine; T2DM, type 2 diabetes mellitus; TDF, tenofovir disoproxil fumarate.

^a^Differences (*P* < .05) in multiple comparisons.

^b^Censored at diagnosis of T2DM or at 31 December 2019 if no T2DM.

The characteristics of this population were similar to those of the overall cohort (Table 3). Follow-up was >33 months in 64% of patients. No significant differences were found for losses to follow-up or global mortality among the patients who developed T2DM and those who did not. The univariable and multivariable analyses showed age and time in education to be associated with T2DM, consistent with the previous model that did not include ART. In addition, having received TDF + (3TC or FTC) + RPV was almost significant as a protective factor (hazard ratio, 0.49; 95% CI, .24–1.01; *P* = .05; Table 4). None of the other 3 combination studies were associated with a diagnosis of T2DM.

**Table 4.** Factors Associated With a Diagnosis of T2DM, Including ART

	Total Cohort				Cohort With Follow-up >33 mo			
	Univariable		Multivariable (n = 4658), HR (95% CI), *P* Value		Univariable		Multivariable (n = 3123), HR (95% CI), *P* Value	
	No.	HR (95% CI), *P* Value	Enter	Final	No.	HR (95% CI), *P* Value	Enter	Final
TDF + (3TC/FTC) + EFV	5983	0.97 (.66–1.43), .876	0.96 (.55–1.64), .868	NS	3895	0.85 (.58–1.24), .397	0.87 (.51–1.50), .617	NS
TDF + (3TC/FTC) + RPV	5983	0.54 (.30–.98), .041	0.49 (.21–1.11), .088	.53 (.25–1.09), .084	3895	0.49 (.27–.89), .018	0.45 (.20–1.03), .059	0.49 (.24–1.01), .054
TDF + (3TC/FTC) + DRV/b	5983	1.05 (.66–1.67), .827	0.64 (.34–1.24), .188	NS	3895	1.00 (.63–1.58), .985	0.62 (.32–1.19), .153	NS
ABC + 3TC + DTG	5983	1.29 (.82–2.03), .277	0.92 (.49–1.73), .808	NS	3895	1.91 (1.22–2.99), .005	1.22 (.65–2.28), .537	NS
Age	5983	1.07 (1.06–1.09), <.001	1.07 (1.05–1.09), <.001	1.07 (1.06–1.09), <.001	3895	1.07 (1.06–1.09), <.001	1.07 (1.05–1.08), <.001	1.07 (1.05–1.09), <.001
HCV before ART	5835	2.34 (1.40–3.90), .001	1.63 (.89–2.98), .112	NS	3797	2.23 (1.38–3.84), .001	1.59 (.87–2.91), .133	NS
AIDS before baseline	5983	2.37 (1.55–5.60), <.001	1.18 (.68–2.05), .564	NS	3895	2.32 (1.53–3.53), <.001	1.15 (.66–2.00), .630	NS
CD4, cells/mL	5809	1.00 (1.00–1.00), .001	1.00 (1.00–1.00), .759	NS	3777	1.00 (1.00–1.00), .002	1.00 (1.00–1.00), .813	NS
Education >12 y	4773	0.49 (.30–.79), .003	0.89 (.57–1.40), .623	.59 (.37–.96), .034	3207	0.49 (.30–.79), .003	0.86 (.55–1.35), .510	0.60 (.37–.97), .038
CV NAE before baseline	5983	5.43 (2.01–14.71), .001	0.64 (.39–1.05), .075	NS	3895	5.38 (1.99–14.6), .001	0.64 (.39–1.05), .079	NS

Abbreviations: 3TC, lamivudine; ABC, abacavir; ART, antiretroviral therapy; CV, cardiovascular non-aids events; DRV, daruanvir/boosted; DTG, dolutegravir; EFV, efavirenz; FTC, emtricitabine; HCV, hepatitis C virus; HR, hazard ratio; NAE, non-aids events; NS, not significant; RPV, rilpivirine; TDF, tenofovir disoproxil fumarate.

## DISCUSSION

We performed a retrospective analysis of the incidence of T2DM after initiation of ART during 2010 to 2019 among PLHIV in a national prospective cohort in Spain. We found an overall cumulative incidence of 5.9 (95% CI, 5.1–6.7) per 1000 person-years of follow-up. This increased significantly to 14.4 (95% CI, 10.4–19.3) in persons aged >50 years. We recorded a median time of 2.5 years to diagnosis of T2DM. The analysis of factors associated with a diagnosis of T2DM revealed that only age and time in education >12 years were significant. Interestingly, time in education >12 years was associated with a 33% reduction in the incidence of T2DM.

Two recent studies analyzed the incidence of and trends in T2DM in PLHIV and attempted to determine whether these were as expected for the general population with similar characteristics or higher. Bratt et al reported 3-fold higher incidence rates (12/1000 person-years) among PLHIV, and Spieler et al reported increased incidence rates from 1.04 per 1000 person-years in 2008 to 1.55 in 2018. Both studies included patients with a median age of 50 years (similar to ours), with differences for sex and race (HR, 2.4 for Black women) and socioeconomic level [5, 16]. This variability in the results obtained highlights the need to determine incidence rates at a more local level so that we can make more accurate predictions with the aim of taking preventive measures or reaching an early diagnosis. Therefore, we propose our study within the framework of a national prospective cohort of PLHIV who were followed up more recently (ie, since 2010) and received more homogeneous care and treatment. We believe that such differentially important aspects in Spain have been well covered in the present analysis, such as the Mediterranean diet, lifestyle associated with the climate, availability of universal health care, and potential genetic factors. It is important to note the considerable role of socioeconomic health determinants that were evident in our study, where time in education was the factor most closely associated with a 70% reduction in the incidence of new cases of T2DM. For many years now in Spain, populations with a lower time in education have been characterized by higher percentages of overweight, obesity, and increased abdominal circumference, all of which are key factors associated with T2DM [17].

Our results for the incidence of T2DM are consistent with those reported in the meta-analysis of Nansseu et al [14], who found an incidence of 8 (95% CI, 5.4–11.2) per 1000 person-years of follow-up in the European population and that age was also a key predictor for development of T2DM. However, studies performed in Spanish, Italian, and French populations in the analysis of Nansseu et al cited T2DM incidence rates of 14 to 28 per 1000 person-years—that is, much higher than those that we report, probably because these studies were published before 2014, when the main drugs used were preintegrase inhibitor regimens based on ritonavir-boosted protease inhibitors (PIs) and the first nucleoside reverse transcriptase inhibitors and nonnucleoside reverse transcriptase inhibitors, which were associated with a higher incidence of T2DM. In their subanalysis of patients with prediabetes, the authors noted a high incidence of 125 (95% CI, 0–423) per 1000 person-years of follow-up, thus highlighting the growing importance of acquisition of this disease among PLHIV in the coming decades. Among the results that we report, we note that the time to onset of a new diagnosis of diabetes, 27 months, is short, given that the patients were aged <50 years. Since prediabetes is the main risk factor for T2DM, we think that the results for new diagnoses of T2DM in our study—which were recorded over a short follow-up period, after integrase inhibitors became available—are consistent with those of a population with a high risk of T2DM at the time of diagnosis of HIV infection.

Another key aspect of the incidence of T2DM in PLHIV is the presence of specific factors, such as HIV itself, chronic activation of the immune system, and the administration of ART drugs from different families with different metabolic toxicity profiles. Some studies, such as those of Ye et al [18] and Tiozzo et al [19], revealed that nadir CD4 lymphocyte count, HIV viral load, and duration of infection are associated with higher incidence rates. However, these associations did not occur in more recent studies [5, 16]. Similarly, we found no relation between factors associated with HIV infection and a new diagnosis of T2DM, most likely because we analyzed a population with a more recent diagnosis of HIV infection who initiated ART early after diagnosis in less metabolically toxic combinations, thus underscoring the importance of inflammation and drug toxicity as potential major predisposing factors for T2DM in PLHIV diagnosed earlier.

As for the role of ART in the development of T2DM, findings are even more striking than for sociodemographic and environmental factors. The main difficulty is the lack of large-scale, well-designed studies aimed at meeting this objective and the difficulty maintaining the same ART regimen over long periods, which considerably hampers more direct analyses. Therefore, we performed a subanalysis of patients who maintained their first ART regimen for at least 2 years—or their second regimen, if the switch was made during the first 6 months—using the 4 preferred ART combinations per the guidelines from 2010 and thereafter, as prescribed to most of our cohort. None of the regimens studied was associated with an increase in the incidence of T2DM. We found an almost significant trend toward a lower incidence of T2DM with the regimen comprising TDF with 3TC or FTC and RPV. To our knowledge, this is the first time that such a trend has been reported. The favorable metabolic profile of RPV-containing regimens is well documented. Furthermore, there have recently been reports of reduced steatosis and liver fibrosis in animals with nonalcoholic fatty liver disease treated with RPV and in individuals who were treated and cured of HCV infection [20–26]. Our results suggest that this benefit could be extended to carbohydrate metabolism, although the hypothesis should be confirmed in other populations. Importantly, we identified RPV-containing regimens to be associated with a lower incidence of T2DM.

No greater risk of T2DM was observed for combinations with a boosted PI or with DTG. After comparing data from other cohorts, we perceived differences with boosted PI regimens because the most widely used PI was boosted darunavir, which clearly has a better metabolic safety profile than first-generation PIs. As for DTG, our results are clear when it is combined mainly with ABC and 3TC and administered mainly to men (85%). Moreover, our study population included practically no Black patients (4%), in whom greater increases in BMI and metabolic syndrome are observed after initiation of DTG-based ART.

Our study is subject to the limitations that are inherent to cohort studies—namely, residual confounding, which is not possible to control for. It is important to note the absence of variables such as body weight and BMI in a high percentage of patients, a family history of diabetes, hemoglobin A1c, genetic factors, and statins. Of note, women accounted for a small percentage of the cohort, thus limiting the conclusions that can be drawn for this subgroup of PLHIV and highlighting the need for cooperative studies of large cohorts in developed countries if we are to further analyze HIV infection and its comorbidity in women. The strengths of our study, however, include the high number of PLHIV studied and the long follow-up period, during which the metabolic toxicity of the nucleoside reverse transcriptase inhibitors and boosted PIs was much less severe than in previous eras, thus making our results more robust and applicable. Furthermore, we believe that the inclusion of socioeconomic variables adds another key dimension in the onset of chronic conditions associated with aging and lifestyle.

In summary, we report a high incidence of T2DM in PLHIV in Spain, especially in persons aged >50 years, with age being the factor most closely associated with onset of T2D and time in education the factor most associated with reduced risk. Our study highlights the lack of association between HIV-related factors and development of T2DM and shows that, within nonnucleoside reverse transcriptase inhibitors, RPV could prove more benign not only in glucose-related metabolic comorbid conditions but also in lipid-related conditions. Given that DTG forms part of preferred initial ART regimens, we consider it relevant that no association occurred between this agent and onset of T2DM in our study population.

T2DM will prove to be a key disease in the progress and aging of PLHIV. Therefore, prevention strategies, early diagnosis (even in the prediabetes phase), early treatment, and closer follow-up will have an enormous impact on the quality of life of PLHIV in the coming decade.

## Supplementary Material

ofae112_Supplementary_Data

## Supplementary Data

Supplementary materials are available at *Open Forum Infectious Diseases* online. Consisting of data provided by the authors to benefit the reader, the posted materials are not copyedited and are the sole responsibility of the authors, so questions or comments should be addressed to the corresponding author.

## Notes

***Acknowledgments.*** We are grateful to Lucía Serrano for the statistical analysis. We acknowledge Thomas O’Boyle for writing assistance.

***Author contributions.*** Conceptualization: M. L. M., C. B. Data curation C. B., N. E., C. M., S. I.-U., L. M.-C., J. M., A. R., J. I. B., M. C.-J., J. J. G. G., and M. L. M. Formal analysis: M. L. M. Supervision: M. L. M. Writing–original draft: M. L. M., C. B., L. M.-C., J. I. B. Writing–review and editing: M. L. M., C. B., L. M.-C., J. I. B., and J. G. G. All authors have read and agreed to the published version of the manuscript.

***Financial support.*** This work was supported by the Ministerio de Sanidad (RD12/0017/0012), integrated in the Plan Nacional de I + D + I and cofunded by the ISCIII-Subdirección General de Evaluación; the Fondo Europeo de Desarrollo Regional (project PI18/011270), funded by Instituto de Salud Carlos III and cofunded by the European Union; CIBER (Consorcio Centro de Investigación Biomédica en Red; CB 2021); the Instituto de Salud Carlos III; the Ministerio de Ciencia e Innovación; the Unión Europea-NextGenerationEU; and the Cohort of the Spanish HIV/AIDS Research Network, funded by the Instituto de Salud Carlos III through the Red Temática de Investigación Cooperativa en SIDA (RIS C03/173, RD12/0017/0018, and RD16/0002/0006) as part of the Plan Nacional R + D + I and cofunded by the ISCIII Subdirección General de Evaluación y el Fondo Europeo de Desarrollo Regional.
