# Supplementary material for: Incidence of Diabetes Mellitus and Associated Factors in the Era of Antiretroviral Drugs With a Low Metabolic Toxicity Profile
Source: Open Forum Infect Dis. 2024 Mar 1;11(4):ofae112. doi: 10.1093/ofid/ofae112 (PMC10981392; doi:10.1093/ofid/ofae112)
Supplement: ofae112_Supplementary_Data [file ofae112_supplementary_data.docx]

Table S1: Baseline characteristics of the subcohort with follow-up ≥33 months (N=5315)

|  |  | | **TOTAL**  **(N= 5135)** | **NO DM**  **(N= 5123)** | **DM**  **(N= 192)** | **p** | |
| --- | --- | --- | --- | --- | --- | --- | --- |
| ***BASELINE VARIABLES*** | | | | | | | |
| Female sex ^(b)^ |  | | 676 (12.7) | 649 (12.7) | 27 (14.1) | 0.58 |  |
| Age ^(a)^ |  | | 36.5 (29.9-44.5) | 36.1 (29.7-43.8) | 46.3 (39-54.4) | <0.001 |  |
| CD4 (cells/µL) ^(a)^ |  | | 355 (207-508) | 358 (210.5-509) | 295.5 (105-449) | <0.001 |  |
| Body mass index ^(a)^ |  | | 23.5 (21.5-25.8) | 23.4 (21.5-25.6) | 26.6 (23.5-29.6) | <0.001 |  |
| Months between diagnosis of HIV and initiation of ART ^(a)^ | | |  | 3.4 (1.3-14.1) | 1.7 (0.8-7.6) | <0.001 |  |
| Route of infection ^(b)^ | MSM | | 3561 (69.2) | 3464 (69.8) | 97 (53.3) |  | (c) |
|  | IDU | | 196 (3.8) | 180 (3.6) | 16 (8.8) |  | (c) |
|  | Heterosexual relations | | 1356 (26.4) | 1293 (26.1) | 63 (34.6) |  | (c) |
|  | Other | | 30 (0.6) | 24 (0.5) | 6 (3.3) | <0.001 | (c) |
| Origin ^(b)^ | Sub-Saharan Africa | | 174 (3.3) | 161 (3.1) | 13 (6.8) |  | (c) |
|  | Spain | | 3264 (61.4) | 3157 (61.6) | 107 (55.7) |  |  |
|  | Europe | | 779 (14.7) | 734 (14.3) | 45 (23.4) |  | (c) |
|  | Latin America/Caribbean | | 977 (18.4) | 956 (18.7) | 21 (10.9) |  | (c) |
|  | North Africa | | 47 (0.9) | 45 (0.9) | 2 (1) |  |  |
|  | Other | | 74 (1.4) | 70 (1.4) | 4 (2.1) | <0.001 |  |
| Longest time in education ^(b)^ | Basic secondary | | 1482 (33.6) | 1402 (33) | 80 (51) |  | (c) |
|  | Pre-university/Trade school | | 1459 (33.1) | 1416 (33.3) | 43 (27.4) |  |  |
|  | University/Higher | | 1470 (33.3) | 1436 (33.8) | 34 (21.7) | <0.001 | (c) |
| HCV before initiating ART ^(b)^ | | |  | 322 (6.6) | 25 (13.5) | 0.001 |  |
| Corticosteroids and/or bronchodilators before ART ^(b)^ | | |  | 25 (0.5) | 2 (1.0) | 0.25 |  |
| Non-AIDS related events before before ART ^(b)^ | | | 356 (6.7) | 347 (6.8) | 9 (4.7) | 0.3 |  |
| Cardiovascular Non-AIDS related events before before ART ^(b)^ | | | 37 (0.7) | 31 (0.6) | 6 (3.1) | <0.01 |  |
| AIDS before ART ^(b)^ | | | 562 (10.6) | 523 (10.2) | 39 (20.3) | <0.001 |  |
| First ART regimen ^(b)^ | 2NRTI+1NNRTI | | 2107 (39.6) | 2040 (39.8) | 67 (34.9) |  |  |
|  | 2NRTI+1PI | | 1182 (22.2) | 1138 (22.2) | 44 (22.9) |  |  |
|  | 2NRTI+1II | | 1735 (32.6) | 1668 (32.6) | 67 (34.9) |  |  |
|  | Other | | 291 (5.5) | 277 (5.4) | 14 (7.3) | 0.44 |  |
| ***FOLLOW-UP ^(d)^ VARIABLES*** | | | | | | | |
| Interruption first ART regimen ^(b)^ |  | 4146 (78) | | 4030 (78.7) | 116 (60.4) | <0.001 |  |
| Time (months) on ART^(a)^ |  | 59.3 (44-78) | | 60 (45-79) | 27 (8-50) | <0.001 |  |
| Death (total) ^(b)^ |  | 68 (1.3) | | 58 (1.1) | 10 (5.2) | <0.001 |  |
| Loss to follow-up (total) ^(b)^ |  | 266 (5.0) | | 255 (5.0) | 11 (5.7) | 0.61 |  |
| Months of follow-up (total) ^(a)^ | | 61 (46.2-80.3) | | 60.9 (46.2-79.8) | 64.8 (40.7-90.1) | 0.44 |  |
| Months until diagnosis of T2DM ^(a)^ | | --- | | --- | 27.5 (8.2-50.9) | --- |  |

^a^ Median (interquartile range)

^b^ N (%)

^c^ Differences (p<0.05) in multiple comparisons

^d^ Censored at diagnosis of T2DM or at 31-12-2019 if no T2DM

Total: including time after the diagnosis of T2DM
